# Metabolites with SARS-CoV-2 Inhibitory Activity Identified from Human Microbiome Commensals

**DOI:** 10.1128/mSphere.00711-21

**Published:** 2021-12-01

**Authors:** Frank J. Piscotta, Hans-Heinrich Hoffmann, Young Joo Choi, Gabriel I. Small, Alison W. Ashbrook, Bimal Koirala, Elizabeth A. Campbell, Seth A. Darst, Charles M. Rice, Sean F. Brady

**Affiliations:** a Laboratory of Genetically Encoded Small Molecules, The Rockefeller University, New York, New York, USA; b Laboratory of Virology and Infectious Disease, The Rockefeller University, New York, New York, USA; c Laboratory of Molecular Biophysics, The Rockefeller University, New York, New York, USA; Hackensack Meridian Health Center for Discovery and Innovation

**Keywords:** biochemistry, molecular biology, virology

## Abstract

The COVID-19 pandemic has highlighted the need to identify additional antiviral small molecules to complement existing therapies. Although increasing evidence suggests that metabolites produced by the human microbiome have diverse biological activities, their antiviral properties remain poorly explored. Using a cell-based SARS-CoV-2 infection assay, we screened culture broth extracts from a collection of phylogenetically diverse human-associated bacteria for the production of small molecules with antiviral activity. Bioassay-guided fractionation uncovered three bacterial metabolites capable of inhibiting SARS-CoV-2 infection. This included the nucleoside analogue N_6_-(Δ^2^-isopentenyl)adenosine, the 5-hydroxytryptamine receptor agonist tryptamine, and the pyrazine 2,5-bis(3-indolylmethyl)pyrazine. The most potent of these, N_6_-(Δ^2^-isopentenyl)adenosine, had a 50% inhibitory concentration (IC_50_) of 2 μM. These natural antiviral compounds exhibit structural and functional similarities to synthetic drugs that have been clinically examined for use against COVID-19. Our discovery of structurally diverse metabolites with anti-SARS-CoV-2 activity from screening a small fraction of the bacteria reported to be associated with the human microbiome suggests that continued exploration of phylogenetically diverse human-associated bacteria is likely to uncover additional small molecules that inhibit SARS-CoV-2 as well as other viral infections.

**IMPORTANCE** The continued prevalence of COVID-19 and the emergence of new variants has once again put the spotlight on the need for the identification of SARS-CoV-2 antivirals. The human microbiome produces an array of small molecules with bioactivities (e.g., host receptor ligands), but its ability to produce antiviral small molecules is relatively underexplored. Here, using a cell-based screening platform, we describe the isolation of three microbiome-derived metabolites that are able to prevent SARS-CoV-2 infection *in vitro*. These molecules display structural similarities to synthetic drugs that have been explored for the treatment of COVID-19, and these results suggest that the microbiome may be a fruitful source of the discovery of small molecules with antiviral activities.

## INTRODUCTION

The rapid global spread of SARS-CoV-2 has brought on a worldwide public health crisis. While the development of vaccines provides reason for optimism, numerous factors, including the persistent prevalence of SARS-CoV-2 across the globe, the continued emergence of new variants, and high rates of vaccine hesitancy, underscore the long-term need for alternative therapeutic modalities to address COVID-19 (1–3). The human microbiome consists of a diverse collection of bacteria inhabiting distinct ecological niches across the human body, including sites of frequent viral infection, such as the gastrointestinal tract, airways, and skin (4). A growing number of reports indicate that small molecules produced by human-associated bacteria can affect not only host cells but also coinhabiting organisms of the microbiome (5, 6). While many studies have focused on identifying microbiota-encoded metabolites that affect either eukaryotic or prokaryotic cells, there are comparatively few examples of commensal metabolites that affect viral infection of host cells (7, 8). Given that bacteria populate body sites that are frequently exposed to viruses, we sought to determine whether commensal bacteria encode metabolites that can inhibit SARS-CoV-2 infection.

Culture broth extracts from 50 phylogenetically diverse and commonly seen commensal bacteria were screened in a cell-based assay for the ability to inhibit infection of human cells by SARS-CoV-2. Bioassay-guided fractionation and structure elucidation of the antiviral activities detected in this extract library led to our identification of three metabolites with anti-SARS-CoV-2 activity. These include the adenosine analogue N_6_-(Δ^2^-isopentenyl)adenosine (IPA), the 5-hydroxytryptamine receptor (5-HTR) agonist tryptamine, and the pyrazine 2,5-bis(3-indolylmethyl)pyrazine (BIP). Interestingly, there are clear structural and functional parallels between commensal bacteria-encoded and synthetic anti-SARS-CoV-2 active small molecules, as synthetic nucleosides, 5-HTR signaling modulators, and pyrazines have all been examined in either COVID-19 clinical trials or observational studies (e.g., remdesivir, fluvoxamine, and favipiravir, respectively) (9–11). The identification of multiple commensal bacteria-encoded metabolites with anti-SARS-CoV-2 activity in this study suggests that the human microbiota likely represents an underexplored reservoir of antiviral small molecules.

## RESULTS

### Creation and screening of a microbiome metabolite library.

**FIG 1:**
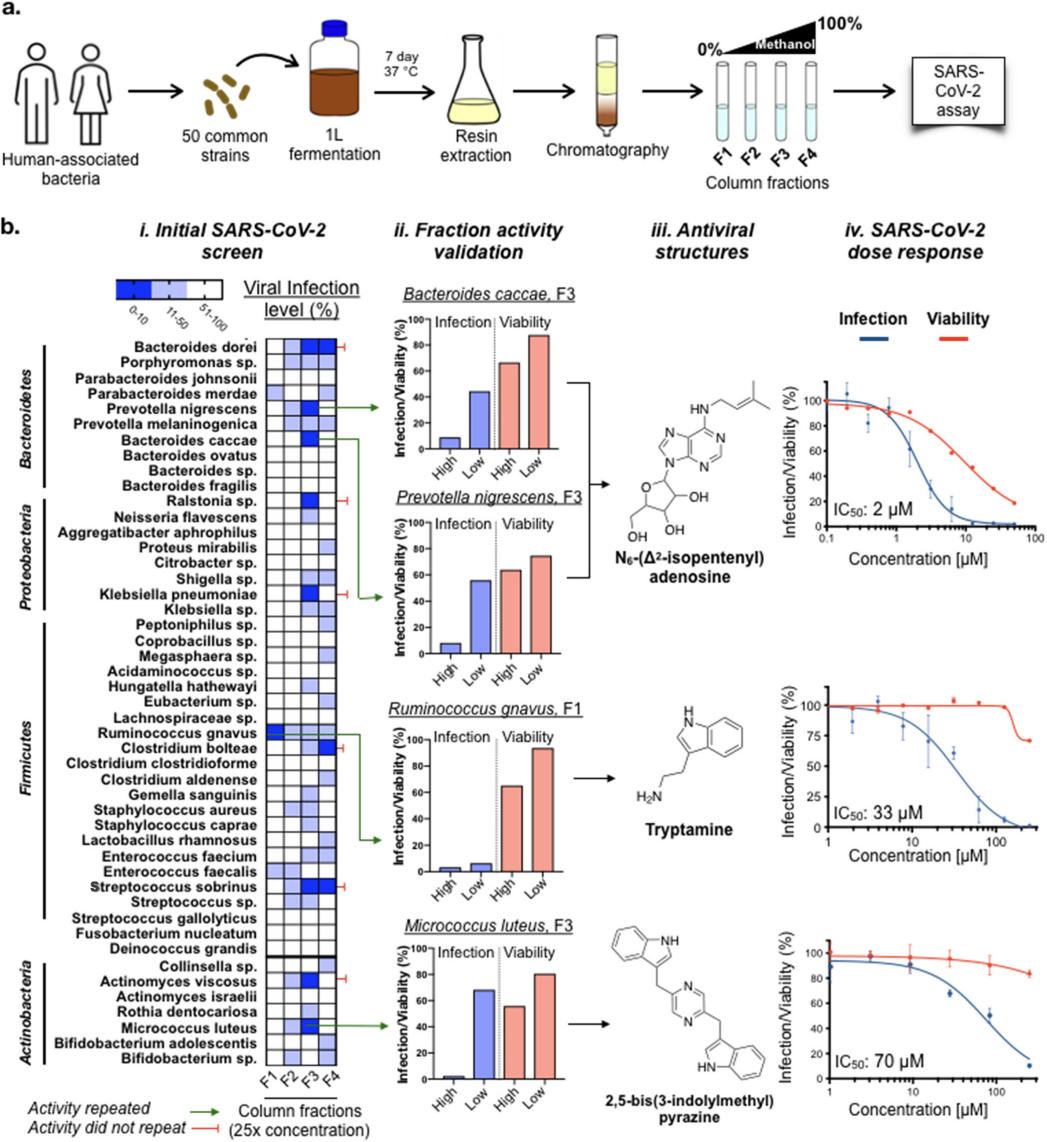
Screening of human-associated bacteria for the production of anti-SARS-CoV-2 active metabolites. (a) Schematic for the construction of a fractionated culture broth extract library and antiviral screening. (b, i) Anti-SARS-CoV-2 activity of fractionated culture broth extracts from 50 human-associated bacteria. *n *= 3. (b, ii and iii) Antiviral active metabolites were isolated from column fractions that showed reproducible antiviral activity. *n *= 3. (b, iv) Anti-SARS-CoV-2 activity and cytotoxicity of each purified metabolite in Huh-7.5 cells. *n = *3; data are mean ± SD.

Although the human microbiome is composed of thousands of unique strains, metabolic redundancy across related bacteria suggests that examining a phylogenetically diverse subset of commensal bacteria is likely to uncover many of the common biologically active molecules that are produced by the microbiota (12). For this study, we selected 50 strains cultured from body sites most likely to come in contact with viruses (e.g., gastrointestinal tract, respiratory tract, and skin) and distributed across the major phyla present in the human microbiome (e.g., *Firmicutes*, *Bacteroidetes*, *Actinobacteria*, and *Proteobacteria*). We sampled species from genera that are highly represented (e.g., *Bifidobacteria*, *Bacteroides*), as well as from those with only a small number of commensal species (e.g., *Megasphaera*, *Coprobacillus*), which allowed us to expand the phylogenetic diversity of our collection. Each strain was grown as a monoculture under the appropriate oxygen environment. After incubating at 37°C for 7 days, extracellular metabolites from these cultures were collected on Amberlite XAD7HP polymeric adsorbent. Resin-bound metabolites were then eluted to yield a library of crude bacterial extracts associated with a diverse collection of human-associated bacteria. In order to facilitate bioactive small-molecule discovery, we performed a prefractionation procedure on each extract in which the crude sample was partitioned by reverse-phase chromatography into 4 subfractions of increasing hydrophobicity (Fig. 1a). The resulting extract fractions were tested at concentrations equivalent to a 25-fold (high) and an 8-fold (low) culture broth concentrate in an *in vitro* cell-based SARS-CoV-2 (USA-WA1/2020) infection assay.

At the higher testing concentration (a 25-fold concentrate of culture broth), 10 bacteria produced at least 1 fraction that reduced viral infection to less than 10% (Fig. 1b). To determine which of these activities were the result of individual antiviral metabolites instead of nonspecific inhibition due to the complex mixture of metabolites in the extract, each fraction that showed >90% inhibition was further partitioned by C-18 semipreparative high-performance liquid chromatography (HPLC), and the resulting fractions were subject to another round of antiviral screening. Extracts from cultures of 4 bacteria (Ruminococcus gnavus, Prevotella nigrescens, Bacteroides caccae, and Micrococcus luteus) generated discrete antiviral-active HPLC fractions, from which individual anti-SARS-CoV-2 metabolite(s) were purified and structurally characterized (Fig. 1b).

### Characterization of microbiome-encoded SARS-CoV-2 active antivirals.

**(i) SARS-CoV-2 active pyrazines.** Partitioning of the anti-SARS-CoV-2 active fraction from a culture of M. luteus SK58 that was isolated from a human skin sample (Human Microbiome Project [HMP] ID 569) (13) revealed a major antiviral active compound and a second HPLC peak with a similar UV trace but only minimal anti-SARS-CoV-2 activity. Structural elucidation of these two compounds by nuclear magnetic resonance (NMR) spectroscopy and high-resolution mass spectrometry (HR-MS) indicated that the primary antiviral compound was 2,5-bis(3-indolylmethyl)pyrazine (BIP) (Fig. S1 in the supplemental material), and the second compound was 2-(3-indolylmethyl)-5-methylpyrazine (IMP) (Fig. S2 and S9C). To confirm these structures, BIP and IMP standards were synthesized from α-amino aldehydes using existing methods for producing pyrazine alkaloids (14). Based on NMR and HR-MS, the synthetic and natural materials were spectroscopically identical (Fig. S3 and S4). In dose-response testing against SARS-COV-2 infection of Huh-7.5 cells, purified BIP had a 50% inhibitory concentration (IC_50_) of 70 μM (Fig. 1b), while IMP displayed much weaker antiviral activity (IC_50_ > 250 μM) (Fig. S5). Neither compound displayed significant cytotoxicity (CC_50_ > 250 μM).

10.1128/mSphere.00711-21.1 FIG S1 ^1^H (600 MHz) and ^13^C (151 MHz) NMR of 2,5-bis(3-indolylmethyl)pyrazine isolated from Micrococcus luteus (DMSO-*d*_6_). Download FIG S1, TIF file, 0.3 MB. Copyright © 2021 Piscotta et al. 2021 Piscotta et al. https://creativecommons.org/licenses/by/4.0/ This content is distributed under the terms of the Creative Commons Attribution 4.0 International license.

10.1128/mSphere.00711-21.2 FIG S2 ^1^H (600 MHz) and ^13^C (151 MHz) NMR of 2-(3-indolylmethyl)-5-methylpyrazine isolated from Micrococcus luteus (DMSO-*d*_6_). Download FIG S2, TIF file, 0.3 MB. Copyright © 2021 Piscotta et al. 2021 Piscotta et al. https://creativecommons.org/licenses/by/4.0/ This content is distributed under the terms of the Creative Commons Attribution 4.0 International license.

10.1128/mSphere.00711-21.3 FIG S3 ^1^H (600 MHz) and ^13^C (151 MHz) NMR of chemically synthesized 2,5-bis(3-indolylmethyl)pyrazine (DMSO-*d*_6_). Download FIG S3, TIF file, 0.3 MB. Copyright © 2021 Piscotta et al. 2021 Piscotta et al. https://creativecommons.org/licenses/by/4.0/ This content is distributed under the terms of the Creative Commons Attribution 4.0 International license.

10.1128/mSphere.00711-21.4 FIG S4 ^1^H (600 MHz) and ^13^C (151 MHz) NMR of chemically synthesized 2-(3-indolylmethyl)-5-methylpyrazine (DMSO-*d*_6_). Download FIG S4, TIF file, 0.3 MB. Copyright © 2021 Piscotta et al. 2021 Piscotta et al. https://creativecommons.org/licenses/by/4.0/ This content is distributed under the terms of the Creative Commons Attribution 4.0 International license.

10.1128/mSphere.00711-21.5 FIG S5 Dose-response curve of IMP for the reduction of SARS-CoV-2 infection in Huh-7.5 cells. Download FIG S5, TIF file, 0.02 MB. Copyright © 2021 Piscotta et al. 2021 Piscotta et al. https://creativecommons.org/licenses/by/4.0/ This content is distributed under the terms of the Creative Commons Attribution 4.0 International license.

10.1128/mSphere.00711-21.9 FIG S9 Dose-response curves of structurally related small molecules of IPA (a), tryptamine (b), and BIP (c) against SARS-CoV-2 infection in Huh-7.5 cells. Download FIG S9, TIF file, 0.1 MB. Copyright © 2021 Piscotta et al. 2021 Piscotta et al. https://creativecommons.org/licenses/by/4.0/ This content is distributed under the terms of the Creative Commons Attribution 4.0 International license.

**(ii) SARS-CoV-2 active tryptamine.** NMR and HR-MS data indicated that the anti-SARS-CoV-2 active metabolite produced by R. gnavus CC55_001C that was isolated from a colonic biopsy specimen (HMP ID 1201) (13) was tryptamine (Fig. S6). Its identity and activity were confirmed using a commercial tryptamine standard. R. gnavus is a well-established producer of tryptamine through the decarboxylation of tryptophan, a reaction that cannot be carried out by the human host (15). It is a member of the microbiota of most children and adults and overrepresented in individuals with inflammatory bowel disease (16).

10.1128/mSphere.00711-21.6 FIG S6 ^1^H (600 MHz) and ^13^C (151 MHz) NMR of tryptamine isolated from Ruminococcus gnavus (DMSO-*d*_6_). Download FIG S6, TIF file, 0.3 MB. Copyright © 2021 Piscotta et al. 2021 Piscotta et al. https://creativecommons.org/licenses/by/4.0/ This content is distributed under the terms of the Creative Commons Attribution 4.0 International license.

**FIG 2:**
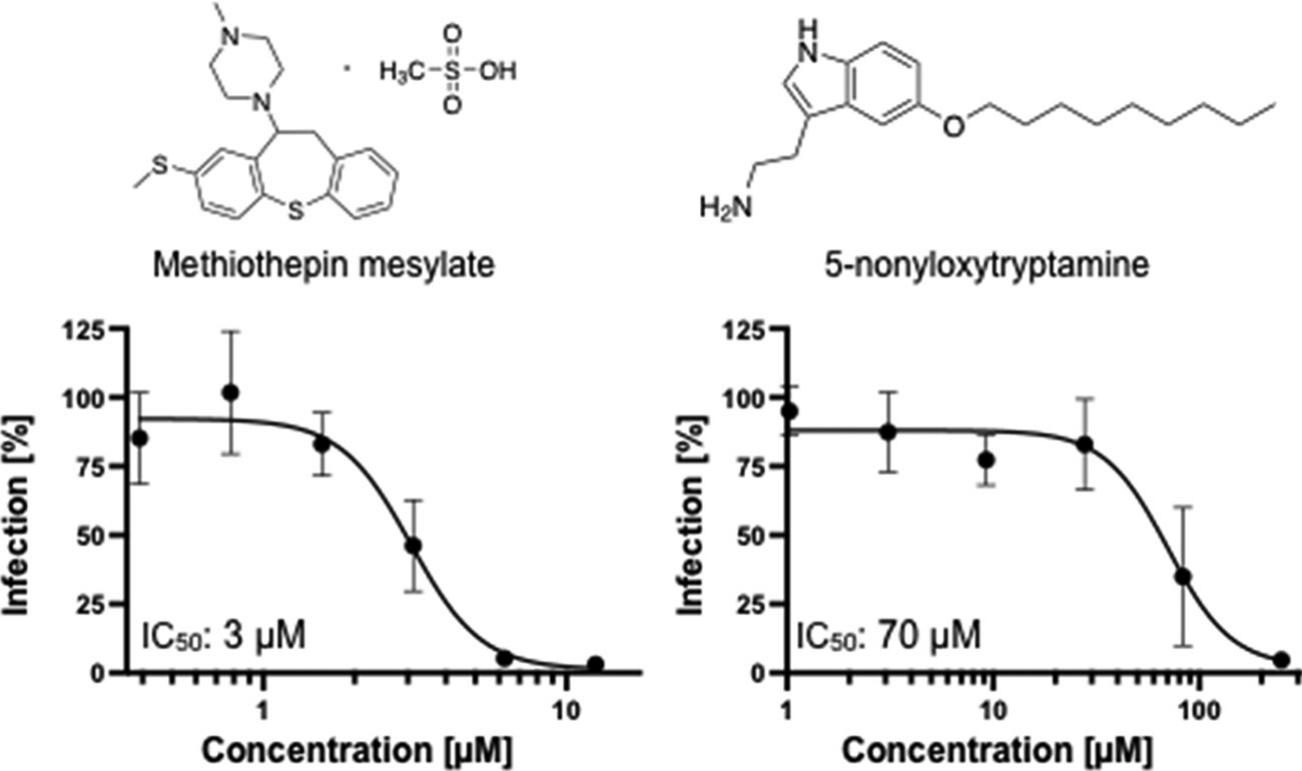
Anti-SARS-CoV-2 activity of 5-HTR signaling modulators. Both the 5-HTR antagonist methiothepin mesylate (left) and agonist 5-nonyloxytryptamine (right) inhibit SARS-CoV-2 infection in Huh-7.5 cells. *n *= 3, data are mean ± SD.

In dose-response testing against SARS-CoV-2 infection of Huh-7.5 cells, tryptamine had an IC_50_ of 33 μM (Fig. 1b), with some loss in cell viability observed at the highest concentration tested (250 μM). Tryptamine is found in human feces at concentrations generally ranging from 10 to 25 μM and as high as 50 μM in some individuals (17), making its ability to inhibit SARS-CoV-2 infection within this same concentration range intriguing. Tryptamine is a potent agonist of a number of serotonin G-protein coupled receptors (GPCRs) (5-hydroxytryptamine receptors [5-HTRs]) and is especially active against HTR2C and HTR5A (50% effective concentration [EC_50_], 70 nM) (5). Although we do not know whether the anti-SARS-CoV-2 activity of tryptamine was due to its 5-HTR agonism, a number of synthetic 5-HTR modulators have been reported to exert antiviral activity against other viruses. For example, the 5-HTR agonist 5-nonyloxytryptamine (5-NT) reduces reovirus entry *in vitro* (18), while the 5-HTR antagonist methiothepin mesylate (MM) was reported to inhibit Chikungunya virus replication (19). To explore the relevance of 5-HTRs modulators to SARS-CoV-2 infection, we assayed MM and 5-NT for anti-SARS-CoV-2 activity. Both MM (IC_50_, 2.8 μM) and 5-NT (IC_50_, 70 μM) exhibited antiviral activity (Fig. 2), illustrating that either agonism or antagonism of 5-HTR receptors can inhibit SARS-CoV-2 infection.

**(iii) SARS-CoV-2 active N_6_-(Δ^2^-isopentenyl)adenosine.** Fractions from extracts of P. nigrescens CC14M that was identified in colonic biopsy specimen (HMP ID 1173) (13) and B. caccae CL03T12C61 cultured from a human fecal sample (HMP ID 1061) (13) yielded SARS-CoV-2 active metabolites with identical HPLC retention times, UV spectra, and masses (*m/z* = 336.1672). Structural elucidation by NMR and HR-MS identified these metabolites as the N_6_-modified adenosine derivative N_6_-(Δ^2^-isopentenyl)adenosine (IPA) (Fig. S7). Comparison of the natural compound to a commercial standard confirmed IPA as the active metabolite produced by both bacteria. Purified IPA inhibits SARS-CoV-2 infection of Huh-7.5 cells with an IC_50_ of 2 μM (Fig. 1b), which is as potent or more potent than a number of repurposed drugs that have been explored as SARS-CoV-2 antivirals (e.g., hydroxychloroquine, IC_50_, 2 μM; ribavirin, IC_50_, 30 μM; and favipiravir, IC_50_ > 100 μM). Notably, IPA also displayed cytotoxicity (50% cytotoxic concentration [CC_50_], 12 μM), something that is often observed with antiviral nucleoside analogues (20).

10.1128/mSphere.00711-21.7 FIG S7 ^1^H (600 MHz) and ^13^C (151 MHz) NMR of *N_6_*-(Δ^2^-isopentenyl)adenosine isolated from Bacteroides caccae (DMSO-*d*_6_). Download FIG S7, TIF file, 0.3 MB. Copyright © 2021 Piscotta et al. 2021 Piscotta et al. https://creativecommons.org/licenses/by/4.0/ This content is distributed under the terms of the Creative Commons Attribution 4.0 International license.

**FIG 3:**
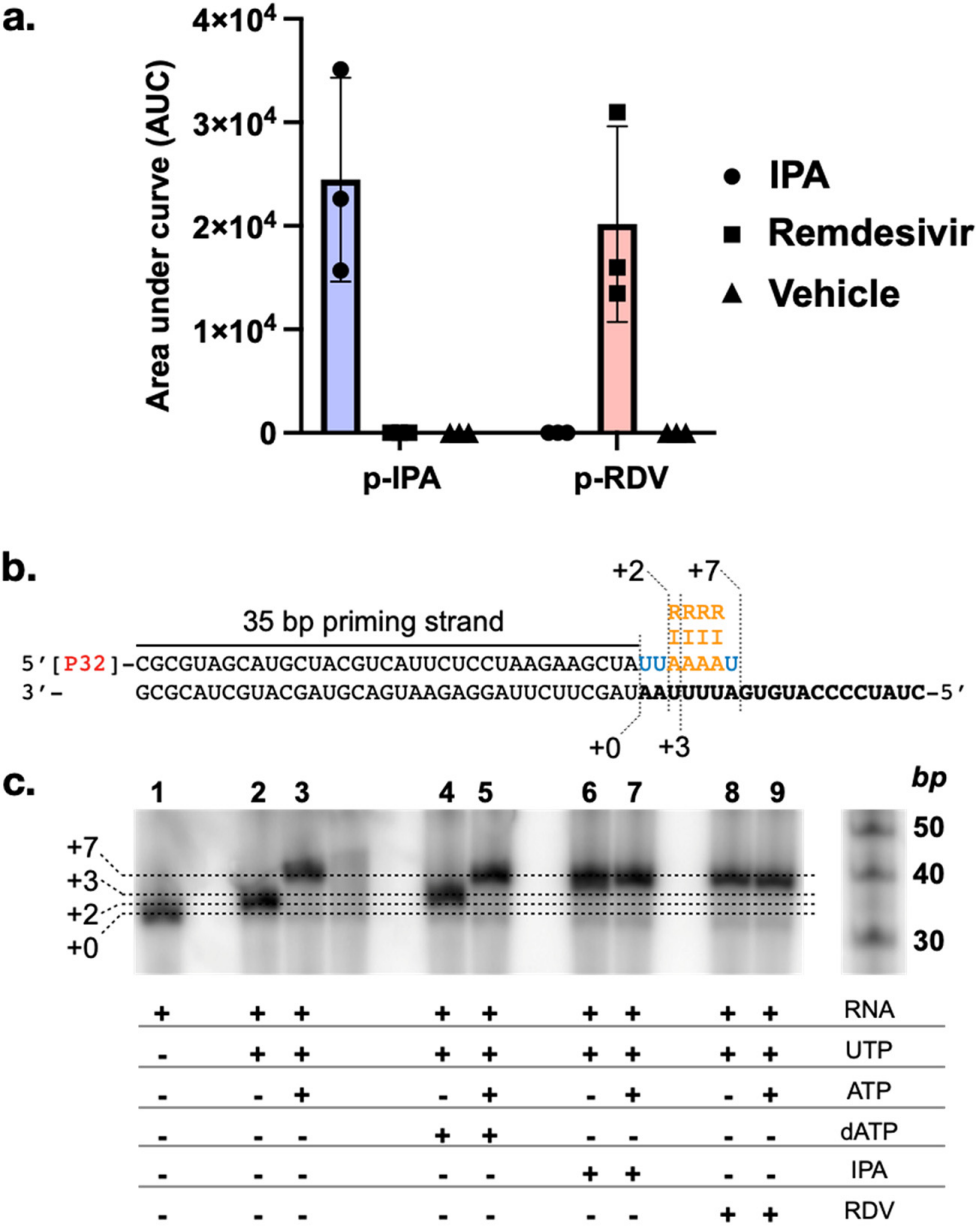
Incorporation of IPA during viral RNA synthesis. (a) Phosphorylation of IPA and remdesivir in HEK293 cells (*n *= 3, data are mean ± SD). (b) Template RNA (black) used to measure the incorporation of ATP (A), IPA 5′-triphosphate (I), or remdesivir 5′-triphosphate (R). (c) RdRp-mediated RNA synthesis in the presence or absence of different nucleoside triphosphates. dATP, 3′-deoxy-ATP.

As IPA was the most potent anti-SARS-CoV-2 metabolite we identified, we sought to probe its anti-SARS-CoV-2 activity in more detail. A common mode of action of nucleoside-based antivirals is incorporation into the product RNA strand by viral RNA-dependent RNA polymerases (RdRp) (21, 22). We, therefore, hypothesized that IPA might be incorporated into RNA by SARS-CoV-2 RdRp. Nucleoside antivirals are prodrugs that must first be phosphorylated *in vivo* in order to function as RdRp substrates (23). The ability of mammalian cells to phosphorylate IPA was examined by incubating HEK293 cells with IPA (10 μM, 3 h) and monitoring the intracellular concentration of the nucleoside and its phosphorylated derivative using negative ionization HR-MS. Similar to the control nucleoside antiviral remdesivir, we observed the appearance of IPA 5′-monophosphate (p-IPA) in IPA-treated cells (Fig. 3a), indicating that IPA, like other nucleoside antivirals, is phosphorylated by eukaryotic cells.

Remdesivir, which is approved for the treatment of severe COVID-19, is efficiently incorporated as an ATP analog into the product RNA strand during SARS-CoV-2 RdRp-catalyzed RNA synthesis (24). The resulting product RNA can then serve as the template strand, which, at intracellular nucleoside concentrations, leads to template-dependent inhibition of SARS-CoV-2 RdRp (25). To test if the SARS-CoV-2 RdRp can incorporate IPA into the product RNA strand, we synthetically triphosphorylated IPA to yield N_6_-(Δ^2^-isopentenyl)ATP (ppp-IPA) (26). *In vitro* RNA synthesis by SARS-CoV-2 RdRp from a template RNA strand (27) (Fig. 3b) was then used to assess the enzyme’s ability to incorporate ppp-IPA. The corresponding triphosphate of remdesivir (ppp-RDV) was used as a positive control. In this assay, the addition of UTP to begin RNA synthesis of a “AAUUUUAG…”-containing template strand resulted in the formation of the +2 product (Fig. 3c, lane 2). Further addition of ATP formed the +7 product (Fig. 3c, lane 3). Addition of the RNA chain terminator 3′-dATP (dATP) forms a +3 product, though the full +7 product could be recovered by the simultaneous addition of ATP (Fig. 3c, lanes 4 to 5). Addition of either ppp-IPA or ppp-RDV resulted in the formation of the +7 RNA product whether ATP was present or not, confirming that ppp-IPA, like ppp-RDV (24), is successfully incorporated into the growing RNA product as an ATP analog by the SARS-CoV-2 RdRp (Fig. 3c, lanes 6 to 9; Fig. S8). IPA may have other targets beyond the viral RdRp. Certain N_6_-modified nucleosides, for example, can interfere with canonical base pairing (28). This provides an alternate mechanism for viral inhibition, with a buildup of mutations resulting in lethal mutagenesis, as seen with other nucleoside antivirals such as ribavirin (29). Mutagenesis as a result of base pair mismatching could also help explain the cytotoxicity exhibited by IPA. Additionally, although nucleoside antivirals are best known for being incorporated by RNA polymerase, other targets, such as RNA capping enzymes, exist (30) and cannot be ruled out as possible targets of IPA. While the incorporation of IPA during SARS-CoV-2-mediated RNA synthesis does not preclude the possibility that it may be imparting antiviral activity through other pathways, it is nonetheless interesting that the naturally occurring nucleoside antiviral IPA can, like the clinically used antiviral remdesivir, be readily incorporated during RNA synthesis.

10.1128/mSphere.00711-21.8 FIG S8 Incorporation of nucleoside derivatives into RNA by the SARS-CoV-2 RdRp. Download FIG S8, TIF file, 1.2 MB. Copyright © 2021 Piscotta et al. 2021 Piscotta et al. https://creativecommons.org/licenses/by/4.0/ This content is distributed under the terms of the Creative Commons Attribution 4.0 International license.

### Structure-activity relationship of microbiome-derived anti-SARS-CoV-2 metabolites.

The structure-activity relationship (SAR) of each anti-SARS-CoV-2 active metabolite was explored using collections of naturally occurring related structures. For IPA, we tested adenine and adenosine, as well as several *N*-modified adenine and adenosine derivatives (Fig. S9a). None of these, including the closely related *trans*-zeatin riboside, displayed antiviral activity (IC_50_ > 250 μM) (Table 1). Although diverse nucleoside structures are used as antivirals, IPA appears to have unique anti-SARS-CoV-2 activity among closely related, naturally occurring nucleosides. We explored the SAR of tryptamine’s anti-SARS-CoV-2 activity using a panel of natural tryptamine analogs, as well as the structurally related trace amines tyramine and phenylethylamine, all of which are known bacterial metabolites (Fig. S9b) (31–33). While both natural (e.g., bufotenine) and synthetic (e.g., 5-NT) tryptamine derivatives have been reported to have antiviral activity (18, 34, 35), among the closely related bacterial metabolites we tested, tryptamine was uniquely active against SARS-CoV-2. 2,5-Disubstituted pyrazines arise from the coupling of two amino acids (36), and thus, the production of a large number of disubstituted pyrazines is possible from proteinogenic amino acids. As the anti-SARS-CoV-2 active pyrazine BIP contained two indole groups, we focused our SAR analysis on testing the relevance of indole to antiviral activity. To do this, we synthesized the phenylalanine-derived analogs of BIP and IMP, 2,5-bis(phenylmethyl)pyrazine (BPP) and 2-(phenylmethyl)-5-methylpyrazine (PMP), and assayed them for anti-SARS-CoV-2 activity. Neither was active in the concentration range we tested (Table 1; Fig. S9c). Once again, the naturally occurring compound we isolated was the most active among the closely related structures we tested.

**TABLE 1:** Anti-SARS-CoV-2 activity of naturally occurring IPA, tryptamine, and BIP structural relatives^*a*^

Structure	IC_50_ (μM)
IPA-related structures	
N_6_-(Δ^2^-isopentenyl) adenosine	2
Adenine	>250
N_6_-(Δ^2^-isopentenyl) adenine	>250
*trans*-Zeatin	>250
Adenosine	>250
N_6_-methyl adenosine	>250
N_6,_N_6_-simethyl adenosine	>250
*trans*-Zeatin riboside	>250
Tryptamine-related structures	
Tryptamine	33
Tryptophan	>250
Indole-3-propionic acid	>250
*N-*Acetyltryptamine	>250
Phenylethylamine	132
Tyramine	>250
Pyrazines	
BIP	70
IMP	>250
BPP	>250
PMP	>250

a *n *= 3; data are mean IC_50_ values.

### Activity against other viruses.

We tested all three microbiome-derived anti-SARS-CoV-2 active metabolites for activity against a panel of RNA viruses, including the seasonal coronavirus HCoV-229E, yellow fever virus 17D (YFV 17D), and human parainfluenza virus 3 (hPIV-3). These are representative of both positive-sense single-strand RNA (+ssRNA) viruses (HCoV-229E, YFV 17D) and negative-sense single-strand RNA (−ssRNA) viruses (hPIV-3). As with SARS-CoV-2, all viral infections were carried out using Huh-7.5 cells.

**FIG 4:**
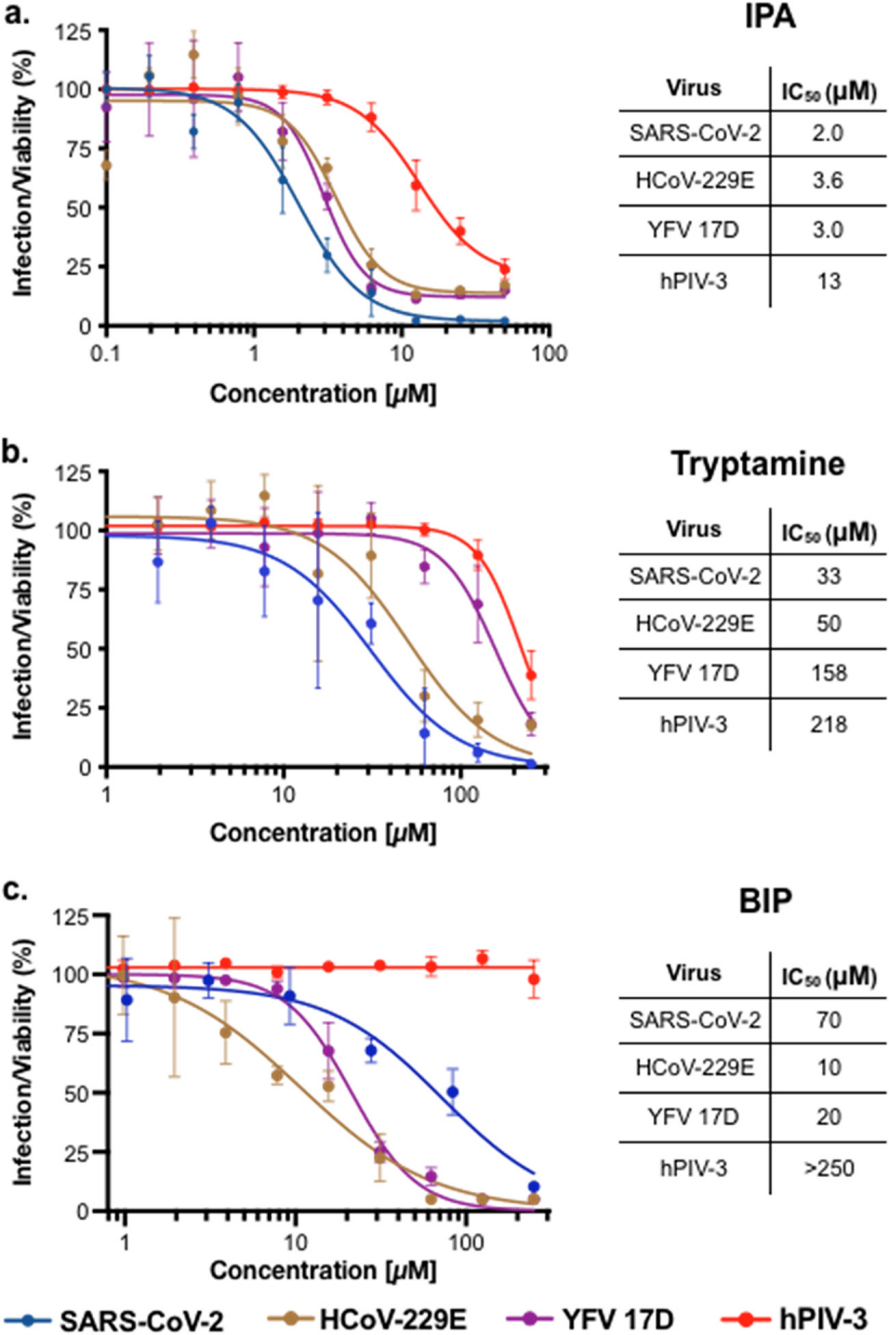
Activity of microbiome-derived antivirals against a panel of RNA viruses. Infection of Huh-7.5 cells by SARS-CoV-2, HCoV-229E, hPIV-3, and YFV 17D was explored. *n *= 3; data are mean ± SD.

IPA displayed broad antiviral activity (IC_50_, 2 to 13 μM). It was most potent against the three +ssRNA viruses (SARS-CoV-2, HCoV-229E, and YFV 17D) with roughly the same IC_50_ (2 μM to 4 μM) against each (Fig. 4a). Although less potent, it also inhibited infection by the lone −ssRNA virus we tested, hPIV-3 (IC_50_, 13 μM). Tryptamine showed a narrower spectrum of activity (Fig. 4b). It was most active against the two coronaviruses, SARS-CoV-2 (IC_50_, 33 μM) and HCoV-229E (IC_50_, 50 μM), and less effective against the −ssRNA virus hPIV-3 (IC_50_ 218 μM). Despite also being a +ssRNA virus, tryptamine was almost five times less active against YFV 17D (IC_50_, 158 μM) than it was against SARS-CoV-2. Like IPA, BIP displayed similar antiviral activity against the three +ssRNA viruses, with IC_50_s of 12, 20, and 70 μM against HCoV-229E, YFV 17D, and SARS-CoV-2, respectively (Fig. 4c). It was inactive against the only −ssRNA virus, hPIV-3. Among IPA, tryptamine, and BIP, tryptamine was unique in its ability to preferably inhibit coronavirus infections.

### Production of anti-SARS-CoV-2 metabolites by commensal strains and the human microbiome.

**FIG 5:**
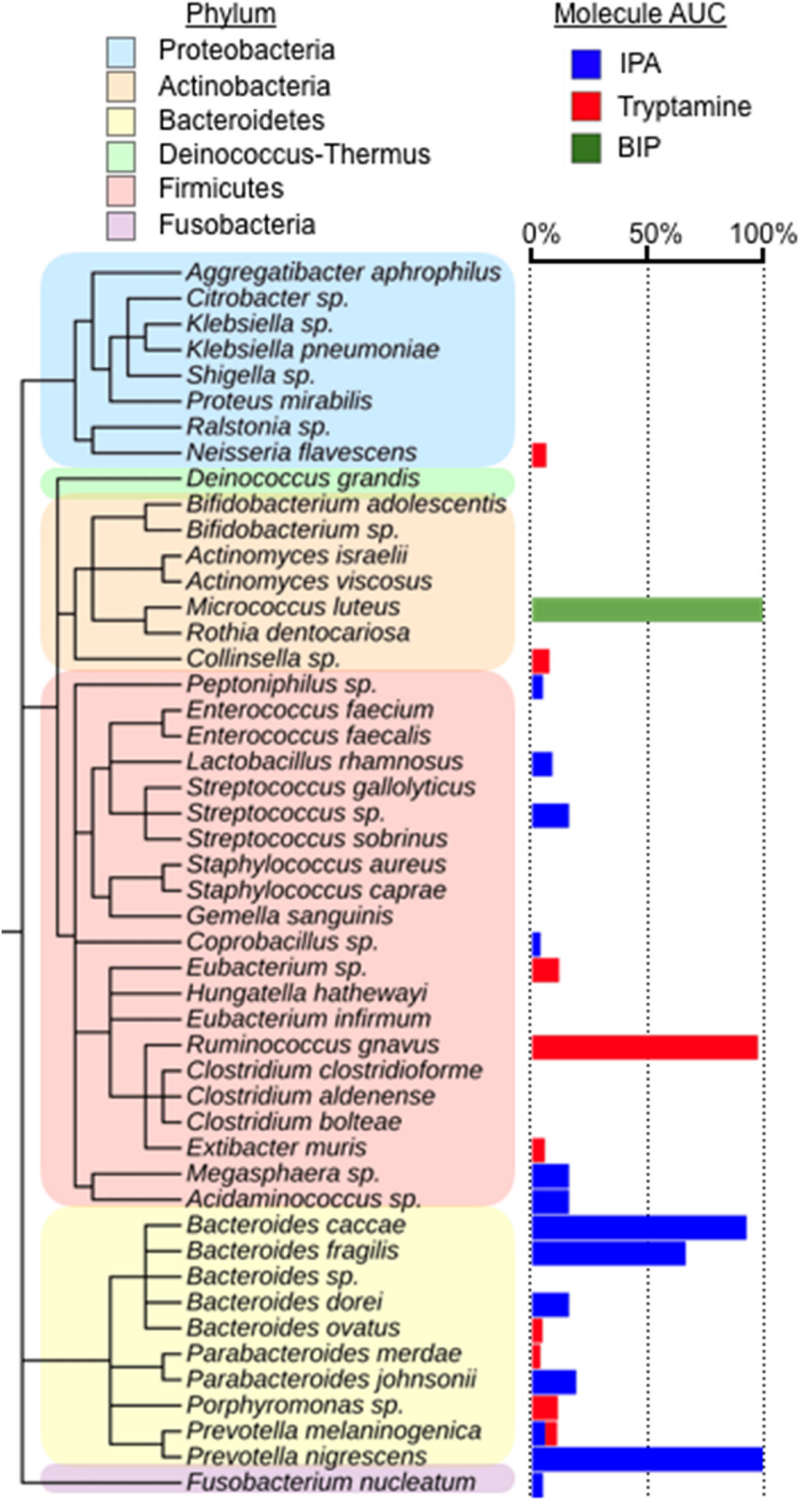
Distribution of anti-SARS-CoV-2 metabolites among culture broth extracts of human-associated bacteria. Production was measured by HR-MS as the area under the curve (AUC) of a metabolite’s parent ion (0% undetected, 100% highest producer). *n = *3. Measurements shown are from a representative culture.

Contrary to what has traditionally been seen in the search for bioactive small molecules from bacteria derived from other ecosystems (e.g., soil), IPA and tryptamine are not specialized metabolites that arise from complex and rare biosynthetic gene clusters. Similar to many of the bioactive molecules that have been characterized from the human microbiota (37, 38), these metabolites instead arise from primary metabolic and catabolic pathways. To look for production of antiviral metabolites by other commensal bacteria, we used HR-MS to track the presence of these metabolites in the 50 culture broth extracts we originally screened for anti-SARS-CoV-2 activity (Fig. 5; Table S1). In the case of IPA, in addition to the two strains from which it was originally characterized, it was also produced in a high titer by Bacteroides fragilis CL07T00C01. IPA was produced at a detectable level most frequently by strains from the *Bacteroidetes* phylum, with half of the *Bacteroidetes* strains we examined producing IPA. The frequent production of IPA by *Bacteroidetes* strains was surprising given that previous reports of bacteria producing high levels IPA, other than as a tRNA constituent, are quite rare (39). Tryptamine is a commonly identified metabolite in human fecal samples (17). Among the strains we examined, it was produced in the highest titer by R. gnavus but was also detected at lower levels in extracts from a number of other bacteria (Fig. 5). Although taxonomically diverse bacteria, including *Bacteroidetes*, *Proteobacteria*, and *Firmicutes* (36, 40, 41), have been reported to produce smaller pyrazine-based natural products, and contrary to IPA and tryptamine, which are produced by a number of bacteria in our collection, BIP and IMP were only produced at detectable levels by M. luteus.

10.1128/mSphere.00711-21.10 TABLE S1 Bacterial strain taxonomy. Download Table S1, XLSX file, 0.02 MB. Copyright © 2021 Piscotta et al. 2021 Piscotta et al. https://creativecommons.org/licenses/by/4.0/ This content is distributed under the terms of the Creative Commons Attribution 4.0 International license.

While tryptamine is ubiquitous in human stool samples, the production of IPA, BIP, and IMP by the human microbiome is not well described. To probe for the *in vivo* production of these metabolites, we looked for tandem mass spectra matching IPA, BIP, or IMP in metabolite extract data from fecal and tissue samples submitted to the GNPS, an open-access metabolomics repository (42). In a number of fecal and tissue samples collected from both mice and humans, we identified tandem mass spectra matching IPA. The detection of IPA in these samples suggests that its production by microbiome-derived bacteria in the laboratory was not merely a by-product of *in vitro* fermentation methods. One human fecal data set was found to contain a spectral match to BIP and IMP. The rarity of these metabolites in stool samples may be explained by a lack of bacterial producers that are native to the gut. Among the strains we examined, the only producing strain, M. luteus, is not found in the gut but, rather, is an aerobic bacterium commonly observed in the skin microbiome (43).

## DISCUSSION

**FIG 6:**
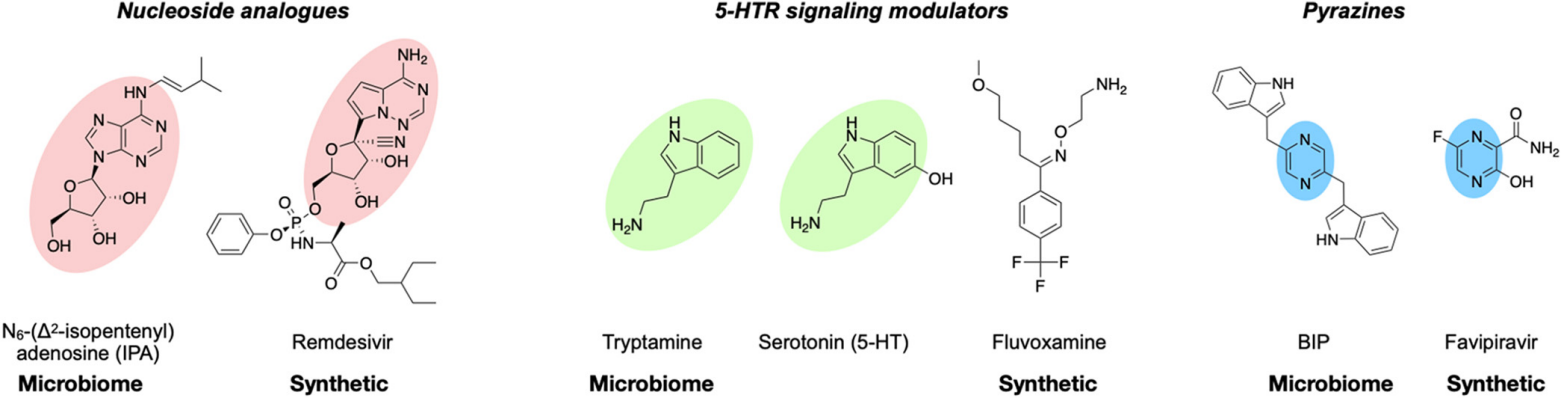
Similarity of microbiome-derived metabolites to synthetic antivirals. Microbiome-derived antiviral metabolites structurally and/or functionally resemble synthetic molecules that have been clinically explored for anti-SARS-CoV-2 activity.

In our survey of culture broth extracts from bacteria isolated from human samples, we identified three structurally diverse metabolites with anti-SARS-CoV-2 activity. Of the metabolites we identified, IPA displayed the most potent antiviral activity, and tryptamine, while less potent, was effective in the concentration range commonly seen in the human gut. All three metabolites are either structurally and/or functionally related to synthetic FDA-approved drugs that have been tested in either COVID-19 clinical trials or observational studies (Fig. 6). IPA is a constituent of bacterial tRNA (39), and very low titers of IPA (4 to 12 μg/liter) have been observed in cultures of a small number of bacterial plant symbiotes (44, 45). Interestingly, P. nigrescens produces more than 500 μg/liter of IPA. Free IPA is best known as a plant cytokinin (46) and has been reported to inhibit enterovirus 71 (EV-71) infection in cell culture (47); however, to the best of our knowledge, neither the production of IPA by human commensal bacteria nor its activity toward SARS-CoV-2 was known.

Tryptamine is a known bacterial agonist of the 5-HTR family of GPCRs. In a small-scale randomized clinical trial, fluvoxamine, a selective serotonin reuptake inhibitor (SSRI) that is typically used to treat obsessive-compulsive disorder, was associated with improved COVID-19 outcomes (9). In addition, in a retrospective study of patients taking antipsychotic and antidepressant serotonin 2A receptor antagonists (e.g., trazadone, mirtazapine), taking these drugs was associated with decreased mortality in severe COVID-19 infections (48). While the mechanisms that explain these observations remain to be determined, the anti-SARS-CoV-2 activity observed for modulators of 5-HTR signaling suggests that it will be interesting to explore the potential antiviral effects of modulating this signaling pathway through engineering of the microbiome.

BIP is a pyrazine-based antiviral produced only by M. luteus among the strains we examined. Small pyrazines like 2,5-dimethylpyrazine have been reported to have antifungal activity, while the larger, indole-containing BIP has been reported to be inactive as an antimicrobial (40, 49). The biosynthesis of small pyrazines is relatively common in nature (50), and opportunistic human pathogens such as Vibrio cholerae and Serratia marcescens have been reported to produce small pyrazines as potential signaling molecules (51, 52). Indole-bearing pyrazines have only been observed a few times in nature (40). In one case, both BIP and IMP were reported as products of a Brevibacillus laterosporus strain that was cultured from the ear of a feral hog (41). The identification of BIP and IMP from M. luteus, a commonly observed human skin commensal, is, to our knowledge, both the first example of indole-containing pyrazines being produced by a human microbiome-derived bacterium and the first report of BIP having antiviral activity. As these metabolites have not been previously reported from the human microbiome, we do not know if they ever accumulate at significant quantities physiologically.

Any potential ecological role of these metabolites in a complex host, microbiome, and virus tripartite interaction remains to be determined. However, the similarity between commensal antiviral metabolites and FDA-approved antivirals is intriguing and unexpected. Our identification of metabolites with anti-SARS-CoV-2 activity suggests that the human microbiota may encode a reservoir of small molecules with antiviral activity and that continued screening of commensal bacterial extracts should be a fruitful strategy for identifying additional antiviral small molecules.

## MATERIALS AND METHODS

### Chemicals, cell lines, and viral strains.

IPA and tryptamine standards were obtained from Cayman Chemical and Alfa Aesar, respectively. All bacterial strains were obtained from BEI Resources, NIAID, NIH, as part of the Human Microbiome Project (13). Huh-7.5 hepatoma cells (Homo sapiens; sex, male; liver epithelial) (53) were cultured in Dulbecco’s modified Eagle medium (DMEM) supplemented with 1% nonessential amino acids (NEAA) and 10% fetal bovine serum (FBS) at 37°C and 5% CO_2_. Cells have been authenticated and tested negative for contamination with mycoplasma. HEK293 cells were maintained in DMEM supplemented with 10% FBS, penicillin/streptomycin, and glutamine.

SARS-CoV-2 (USA-WA1/2020; BEI Resources) and HCoV-229E were amplified and titrated by standard plaque assay (PA) in Huh-7.5 cells (54). The generation of viral stocks for additional viruses has been previously described as follows: hPIV-3 (55) (based on strain JS) and YFV 17D Venus (56) (derived from YF17D-5′C25Venus2AUbi).

### Bacterial fermentations.

All bacteria were cultivated in LBM media (17 g/liter brain heart infusion, 5 g/liter yeast extract, 200 mg MgSO_4_·7H_2_O, 100 mg/liter MnCl_2_·4H_2_O, 5 mg/liter hemin, 1 g/liter maltose, 1 g/liter cellobiose, and 0.5 g/liter l-cysteine). Bacterial fermentations were performed aerobically or anaerobically for each individual strain. For aerobic fermentations, the bacterial glycerol stock provided by BEI was used to inoculate 10 ml of LBM. This culture was grown for 24 to 48 h at 37°C with shaking at 200 rpm, and once, turbid was used to inoculate 1 liter of fresh LBM media in a 3-liter baffled Fernbach flask. The culture was fermented for 7 days at 37°C with shaking at 200 rpm, followed by metabolite extraction as described below. All anaerobic fermentations were performed in a 37°C incubator placed inside vinyl anaerobic chamber (Coy) with a gas mix of 5% CO_2_, 5% H_2_, and 90% N_2_. The bacterial glycerol stock provided by BEI was used to inoculate 10 ml of LBM. This culture was grown for 24 to 48 h, and once, turbid was used to inoculate 1 liter of fresh LBM media in a 1-liter glass bottle (Chemglass). Fermentation proceeded anaerobically for 7 days at 37°C followed by metabolite extraction as described below.

### Small molecule extraction and prefractionation.

Amberlite XAD7HP resin (Sigma) was activated by soaking in methanol for 15 min, followed by washing five times with deionized water. Resin was stored in water until needed. Twenty grams of activated Amberlite resin was added to 1 liter of a cell suspension of bacterial culture and incubated with shaking (200 rpm) at room temperature for 4 h. Resin was then collected by filtration. We added 300 ml of acetone (Sigma), and this mixture was shaken for 2 h at room temperature. The acetone elution was collected, and an equal volume of fresh acetone was added and allowed to incubate with the resin overnight at room temperature. This second acetone elution was combined with the first and dried *in vacuo* on a rotary evaporator (Buchi) to afford a crude metabolite extract.

Each extract was resuspended in methanol (Sigma) to a concentration of 100 mg/ml. One milliliter (100 mg) of this solution was aliquoted and diluted in water to 5% methanol and spun down to prepare for fractionation on a Strata C_18_ solid-phase extraction (SPE) cartridge (500 mg, 3 ml; Phenomenex). The SPE cartridge was activated with two column volumes of methanol followed by washing with two column volumes of water. The crude extract was then passed over the column, and the flowthrough (fraction F1) was collected. Metabolites were then eluted (two column volumes) using a stepwise gradient of increasing methanol concentrations as follows: F2, 30%; F3, 70%; and F4, 100%. These elutions, along with the column flowthrough, were dried *in vacuo*, affording four prefractionated extracts from each crude metabolite pool.

### SARS-CoV-2 infection assay.

The day prior to infection, Huh-7.5 cells were seeded at 7.5 × 10^3^ cells/well into 96-well plates. The next day, serially diluted compounds were added to the wells, followed 1 h later by infections with either SARS-CoV-2 (multiplicity of infection [MOI], 0.05 PFU/cell), HCoV-229E (MOI, 0.05 PFU/cell), YFV 17D Venus (MOI, 0.025 PFU/cell), or hPIV-3-GFP (MOI, 0.05 IU/cell). Cells were incubated for 48 h at 33°C for infections performed with SARS-CoV-2 and at 37°C for infections performed with other viruses. Cells were then fixed by adding an equal volume of 7% formaldehyde to the wells and subsequently permeabilized with 0.1% Triton X-100 for 10 min. After extensive washing, SARS-CoV-2- and HCoV-229E-infected cells were incubated for 1 h at room temperature with blocking solution of 5% goat serum in phosphate-buffered saline (PBS) (catalog no. 005-000-121; Jackson ImmunoResearch). A rabbit polyclonal anti-SARS-CoV-2 nucleocapsid antibody (catalog no. GTX135357; GeneTex) was added to the cells at 1:1,000 dilution in blocking solution and incubated at 4°C overnight. J2, a mouse monoclonal anti-double-stranded RNA (dsRNA) antibody (catalog no. 10010500; Scicons) was added to the cells under similar conditions to detect HCoV-229E-infected cells. Goat anti-rabbit Alexa Fluor 594 (catalog no. A-11012; Life Technologies) and goat anti-mouse Alexa Fluor 488 (catalog no. A-11001; Life Technologies) were used as secondary antibodies at a dilution of 1:2,000. Nuclei for all infections, including those with the fluorescently labeled YFV 17D and hPIV-3, were stained with Hoechst 33342 (catalog no. 62249; Thermo Scientific) at 1 μg/ml. Next, images were acquired with an ImageXpress XLS Widefield High-Content microscope (Molecular Devices, Sunnyvale, CA). Images were acquired using the DAPI (4′,6-diamidino-2-phenylindole) filter (excitation, 377 nm, and emission, 447 nm) for Hoechst staining and the fluorescein isothiocyanate (FITC) filter (excitation, 482 nm, and emission, 536 nm) for viral nucleocapsid staining. Images were analyzed using an automated custom module in the MetaXpress software (Molecular Devices). Default software analysis scripts were used for DAPI- and FITC-positive cell counting. Infection was calculated as the percentage of viable cells that were positive for viral nucleocapsid. For determining the efficacy of isolated metabolites, 10-point dose-response curves in a 1:3 dilution series were tested in triplicate using a maximum concentration of 50 μM (IPA) or 250 μM (tryptamine, BIP, and IMP). IC_50_ values were calculated using a variable-slope, four-parameter nonlinear regression curve fit (GraphPad Prism 8). All SARS-CoV-2 experiments were performed in a biosafety level 3 laboratory.

### Chromatography of bacterial metabolite fractions.

Bacterial metabolite fractions were partitioned on an Agilent Series 1200 HPLC using a solvent system of water (A) and acetonitrile (B; each with 0.1% formic acid) connected to a Waters XSelect CSH phenyl-hexyl OBD prep column (150 mm by 10 mm, 130 Å, 5 μm) run at a constant flow rate of 3 ml/min. The specific chromatographic method was chosen according to the identity of the prefraction as follows: for F1, 0 to 5 min, 5% B; 5 to 10 min, increase to 10% B; 10 to 20 min, 10% B; 20 to 40 min, increase to 30% B; 40 to 45 min, increase to 95% B; 45 to 55 min, 95% B; for F3, 0 to 5 min, 10% B; 5 to 30 min, increase to 30% B; 30 to 40 min, increase to 9% B; 40 to 50 min, 95% B; and for F4, 0 to 5 min, 30% B; 5 to 30 min, increase to 95% B; and 30 to 40 min, 95% B. Fractions were collected in 2-min intervals and dried *in vacuo*.

### Metabolite characterization.

All high-resolution mass spectrometry (HR-MS) data were collected on a Sciex X500B quadrupole time of flight (QTOF) mass spectrometer running in positive mode, using a 5,500-V spray voltage and 5 V collision energy. The mass spectrometer was connected to an Exion liquid chromatography system using water (A) and acetonitrile (B) solvents, each with 0.1% formic acid. Samples were run on a Kinetex PS C_18_ column (50 mm x 2.1 mm, 100 Å, 2.6 μm) at 0.50 ml/min with the following method: 0 to 0.3 min, 10% B; 0.3 to 3.3 min, increase to 95% B; and 3.3 to 3.8 min, 95% B. All analyses were performed on SCIEX OS software. NMR data were collected on a Bruker Avance DMX 600 MHz spectrometer.

### (i) Isopentenyl adenosine.

IPA was isolated from Bacteroides caccae F3 as a single metabolite using the HPLC method described above. [M+H]^+^; calculated, 336.1672; observed, 336.1670. ^1^H NMR (DMSO-*d_6_*, 600 MHz): δ_H_ 8.34 (s, 1H), 8.21 (s, 1H), 7.92 (s, 1H), 5.88 (d, *J *= 6.1 Hz, 1H), 5.43 − 5.40 (m, 2H), 5.30 (t, *J *= 6.7 Hz, 1H), 5.17 (d, *J *= 4.6 Hz, 1H), 4.61 (q, *J *= 5.8 Hz, 1H), 4.14 (q, *J *= 4.2 Hz, 1H), 4.06 (s, 2H), 3.96 (q, *J *= 3.4 Hz, 1H), 3.67 (dt, *J *= 12.1, 4.1 Hz, 1H), 3.55 (ddd, *J *= 11.6, 7.3, 3.6 Hz, 1H), 1.70 (s, 3H), 1.67 (s, 3H). ^13^C NMR (DMSO*-d_6_*, 151 MHz): δ_C_ 154.8, 152.8, 148.7, 140.2, 133.8, 122.5, 120.3, 88.5, 86.4, 74.0, 71.1, 62.2, 25.8, 18.3.

### (ii) Tryptamine.

Tryptamine was isolated from Ruminococcus gnavus F1 as a single metabolite using the HPLC method described above. [M+H]^+^; calculated, 161.1079; observed, 161.1075. ^1^H NMR (DMSO*-d_6_*, 600 MHz): δ_H=_ 10.99 (s, 1H), 8.09 (s, 2H), 7.56 (d, *J *= 7.9 Hz, 1H), 7.36 (d, *J *= 8.1 Hz, 1H), 7.23 (s, *J *= 2.4 Hz, 1H), 7.09 (t, *J *= 7.5 Hz, 1H), 7.00 (t, *J *= 7.4 Hz, 1H), 3.03 (dp, *J *= 12.1, 6.6, 6.1 Hz, 4H). ^13^C NMR (DMSO-*d_6_*, 151 MHz): δ_C_ 136.8, 127.3, 123.8, 121.6, 118.9, 118.6, 112.0, 109.9, 23.5.

### (iii) BIP.

BIP was isolated from Micrococcus luteus F3 as a single metabolite using the HPLC method described above. [M+H]^+^; calculated, 339.1610; observed, 339.1600. ^1^H NMR (DMSO*-d_6_*, 600 MHz): δ_H_ 10.87 (s, 2H), 8.45 (s, 2H), 7.44 (d, *J *= 7.8 Hz, 2H), 7.32 (d, *J *= 8.1 Hz, 2H), 7.18 (s, 2H), 7.04 (t, *J *= 7.5 Hz, 2H), 6.91 (t, *J *= 7.6 Hz, 2H), 4.16 (s, 4H). ^13^C NMR (DMSO*-d_6_*, 151 MHz): δ_C_ 154.2, 143.4, 136.7, 127.3, 123.9, 121.5, 118.9, 112.1, 111.9, 31.3.

### (iv) IMP.

IMP was isolated from Micrococcus luteus F3 as a single metabolite using the HPLC method described above. [M+H]^+^; calculated, 224.1188; observed, 224.1176. ^1^H NMR (DMSO*-d_6_*, 600 MHz): δ_H_ 10.90 (s, 1H), 8.35 (s, 1H), 8.32 (s, 1H), 7.48 (d, *J *= 7.9 Hz, 1H), 7.34 (d, *J *= 8.1 Hz, 1H), 7.21 (s, 1H), 7.05 (t, *J *= 7.5 Hz, 1H), 6.94 (t, *J *= 7.4 Hz, 1H), 4.18 (s, 2H), 2.46 (s, 3H). ^13^C NMR (DMSO*-d_6_*, 151 MHz): δ_C_ 156.0, 152.9, 141.8, 136.7, 127.3, 124.0, 121.5, 118.9, 118.9, 111.9, 31.8, 21.6.

### Chemical synthesis of pyrazines.

**(i) Formation of Weinreb amide.** Benzyloxy carbonyl (Cbz)-protected amino acid (500 mg, 1 equivalent [equiv]) dissolved in 10 ml dimethylformamide (DMF) was mixed with *N*,*O*-dimethylhydroxylamine (2 equiv.) and *N*,*N*-diisopropylethylamine (3 equiv.) and stirred for 1 h at room temperature. The reaction mixture was diluted with dichloromethane (DCM, 30 ml) and washed several times with acidified water (1% formic acid) to remove DMF. DCM was concentrated *in vacuo* and purified by flash chromatography using ethyl acetate-hexane as eluent to give the Weinreb amide as a yellow oil.

**(ii) Formation of aldehyde derivative.** Lithium aluminum hydride (5 equiv.) was added and stirred for 2 h into a stirred solution of Weinreb amide (447 mg, 1 equiv.) in diethyl ether (50 ml) at 0°C. The reaction mixture was quenched with ice cubes, filtered through a celite pad, and washed with water (50 ml) and diethyl ether (30 ml). The filtrate was extracted 3 times with diethyl ether and washed with hydrochloric acid (1 M), saturated sodium hydrogen carbonate solution, and brine. The extract was dried over magnesium sulfate, concentrated *in vacuo*, and purified by flash chromatography using ethyl acetate-hexane as eluent to give the aldehyde derivative as a yellow oil.

**(iii) Formation of pyrazine.** Pd(OH)_2_ (2% on carbon, 10 mg) was added and stirred for 2 h at room temperature under a hydrogen atmosphere into the aldehyde derivative (100 mg) dissolved in a mixture of acetic acid, methanol, and DCM (1:2:2, 5 ml). The hydrogen source was removed, and the reaction mixture was stirred overnight while open to air. The mixture was then filtered through a celite pad, washed with methanol, dried *in vacuo*, and purified by flash chromatography using ethyl acetate-hexane as an eluent. Identities of the desired pyrazines were verified and characterized by HR-MS and NMR.

### Nucleoside phosphorylation in cell culture.

Confluent HEK293 cells were detached with trypsin-EDTA (0.25%) and adjusted to a concentration of 3.0 × 10^6^ cells/ml. Each well of a 6-well plate was seeded (3.0 × 10^6^ cells), and cells were allowed to attach overnight. The medium was aspirated and replaced with 1 ml of fresh DMEM containing either IPA or remdesivir at 10 μM. After 3 h, cells were harvested. The medium was removed, and plates were immediately placed on ice. Ice-cold Hanks’ balanced salt solution (HBSS) was used to wash each well twice, and then, 500 μl extraction solution (80% CH_3_OH/H_2_O, 5% formic acid, and 10 μM oligomycin standard, prechilled at −80°C for >1 h) was added to each well. Cells were allowed to lyse for 15 min on ice. Each well was then scraped with a sterile cell scraper, and the lysate was transferred to an Eppendorf tube. Lysates were centrifuged at 20,000 × *g* for 10 min at 4°C. Supernatants were transferred into preweighed 4-ml glass vials and dried *in vacuo* without heat (CentriVap; Labconco). Dried material was resuspended in 200 μl 50:25:25 H_2_O/CH_3_CN/CH_3_OH and centrifuged at 16,000 × *g* for 10 min at 4°C. Supernatants were moved into 96-well polypropylene plates, covered with slit silicone mats, and stored at −20°C until analysis by HR-MS.

### Chemical synthesis of nucleoside triphosphates.

Trimethyl phosphate (0.5 ml, stored over 4-Å molecular sieves) (0.5 ml) was added to IPA (0.05 mmol) over 4-Å molecular sieves. The mixture was stirred for 24 h under an argon atmosphere. Phosphorus oxychloride (0.0070 ml, 0.075 mmol) was added via syringe, and the mixture was stirred for 3 h at 0°C. A mixture of tributylamine (0.060 ml, 0.25 mmol) and tributylammonium pyrophosphate (0.25 mmol, 90.8 mg) dissolved in acetonitrile (0.25 ml) was added, and the mixture was stirred for an additional 30 min at 0°C. The reaction was quenched by addition of triethylammonium bicarbonate (TEAB) (1 M) (0.5 ml) and water (5 ml). The reaction mixture was purified by HPLC using the conditions described above with the following solvent gradient: 0 to 4 min, 5% B; 4 to 30 min, increase to 60% B; 35 to 40 min, increase to 95% B; and 40 to 50 min, 9% B. The identity of the IPA 5′-triphosphate was confirmed by HR-MS.

### RNA incorporation by SARS-CoV-2 RdRp.

RNA primer (5′-CGCGUAGCAUGCUACGUCAUUCUCC UAAGAAGCUA-3′) was labeled at the 5′ end with T4 polynucleotide kinase (New England Biolabs) and [γ^32^-P]ATP (PerkinElmer). The labeling reaction was heat inactivated at 70°C for 20 min, and unincorporated [γ^32^-P] ATP was removed with a 7 molecular weight cutoff (MWCO) Zeba spin column (Thermo Fisher). The labeled RNA primer was then annealed to the template strand (5′-CUAUCCCCAUGUGAUUUUAAUAGCUUCUUAGGAGAAUGACGUAGCAUGCUACGCG-3′; Horizon Discovery) by heating to 95°C for 2 min and cooling to 10°C for 30 min. Proteins of the SARS-CoV-2 replication-transcription complex were purified as previously reported (27, 57). Nsp12 was incubated with 2.5-fold molar excess of Nsp7 and Nsp8 at room temperature for 15 min and buffer exchanged into transcription buffer (20 mM HEPES, pH 8.0, 10 mM K-acetate, 2 mM MgCl_2_, and 1 mM dithiothreitol [DTT]) through a 7 MWCO Zeba Spin desalting column (Thermo Fisher). The holo-complex (1.25 μM) was incubated with the RNA scaffold (1 μM) at 37°C for 15 min before the addition of NTPs and inhibitors (50 μM) 3′-deoxyadenosine-5′-triphosphate (TriLink), remdesivir triphosphate (Gilead Sciences), and isopentenyl ATP in 10-μl reactions. *In vitro* transcription reaction mixtures were incubated at 30°C for 10 min before being quenched with an equal volume of 2× stop buffer (95% [vol/vol] formamide, 20 mM EDTA, 0.05% bromophenol blue, and xylene cyanol). Elongation products were separated on 15% acrylamide-6 M urea denaturing gel alongside Decade RNA ladder (Thermo Fisher) in 1× Tris-borate-EDTA (TBE) and analyzed by phosphorimaging.

### Data availability.

All data that support the findings of this study are available from the corresponding author upon request.
