# Supplementary figures and images for: Metabolites with SARS-CoV-2 Inhibitory Activity Identified from Human Microbiome Commensals
Source: mSphere. 2021 Dec 1;6(6):e00711-21. doi: 10.1128/mSphere.00711-21 (PMC8636105; doi:10.1128/mSphere.00711-21)

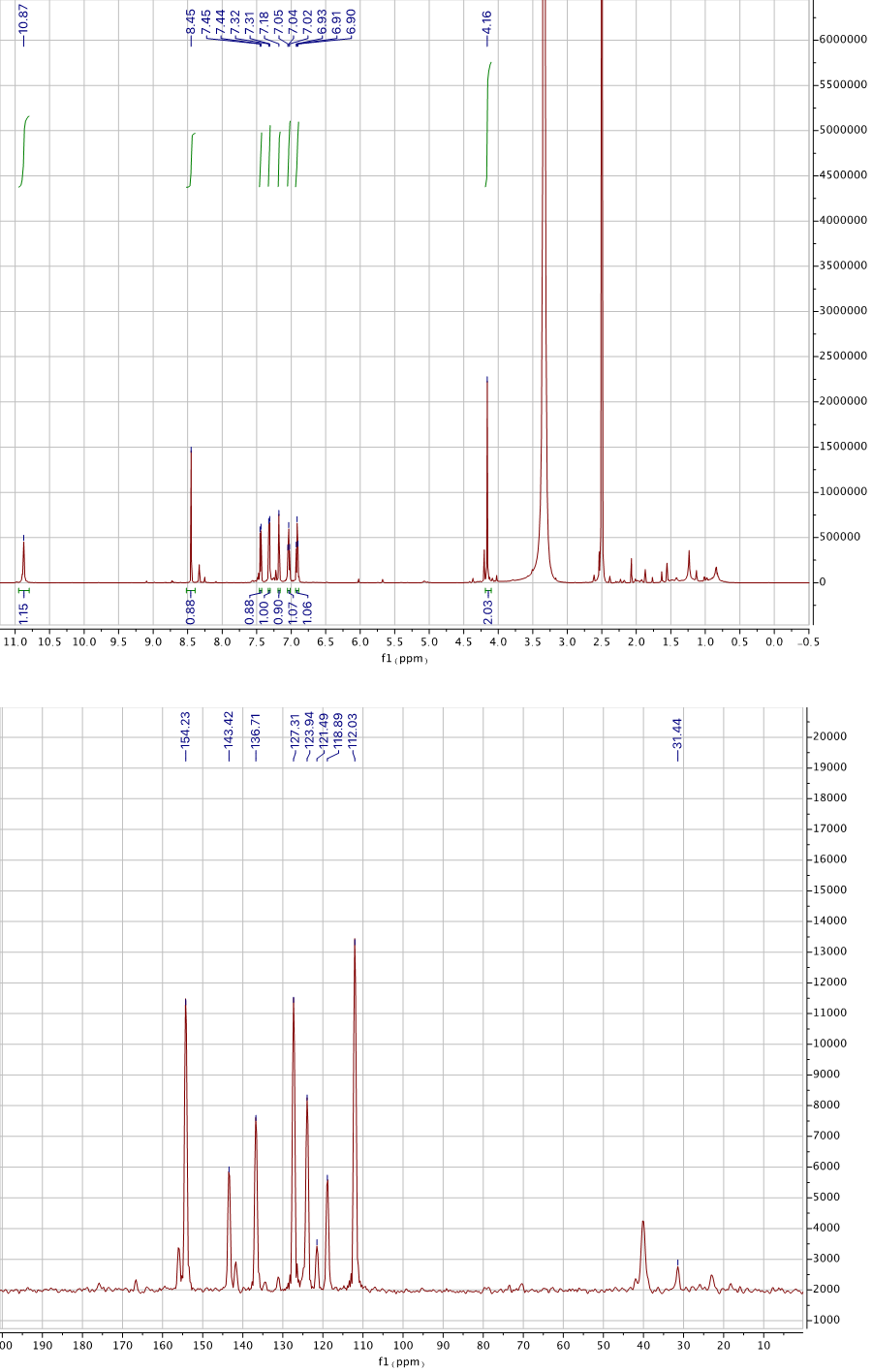

Supplement: FIG S1 [file msphere.00711-21-sf001.tif]

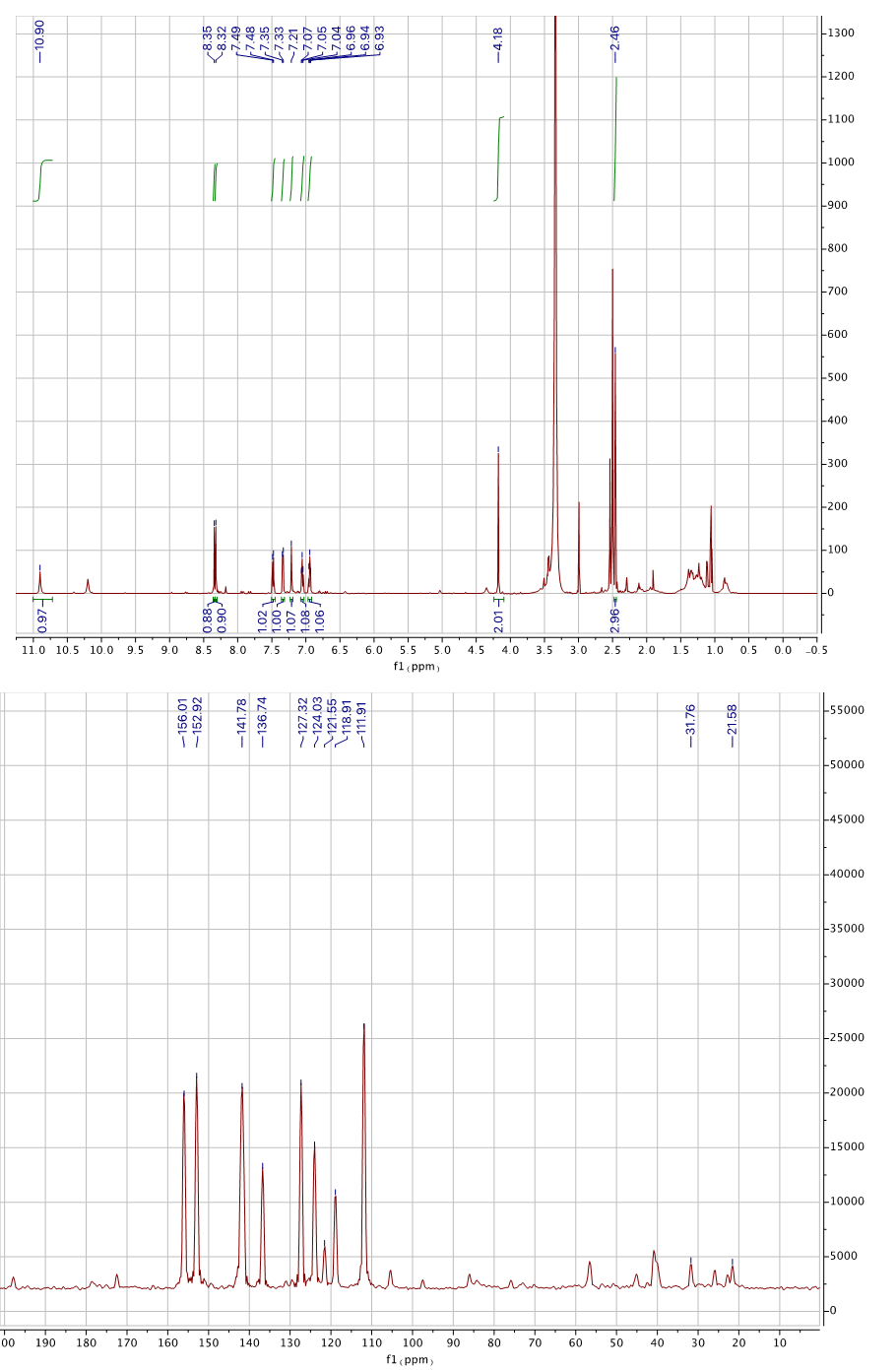

Supplement: FIG S2 [file msphere.00711-21-sf002.tif]

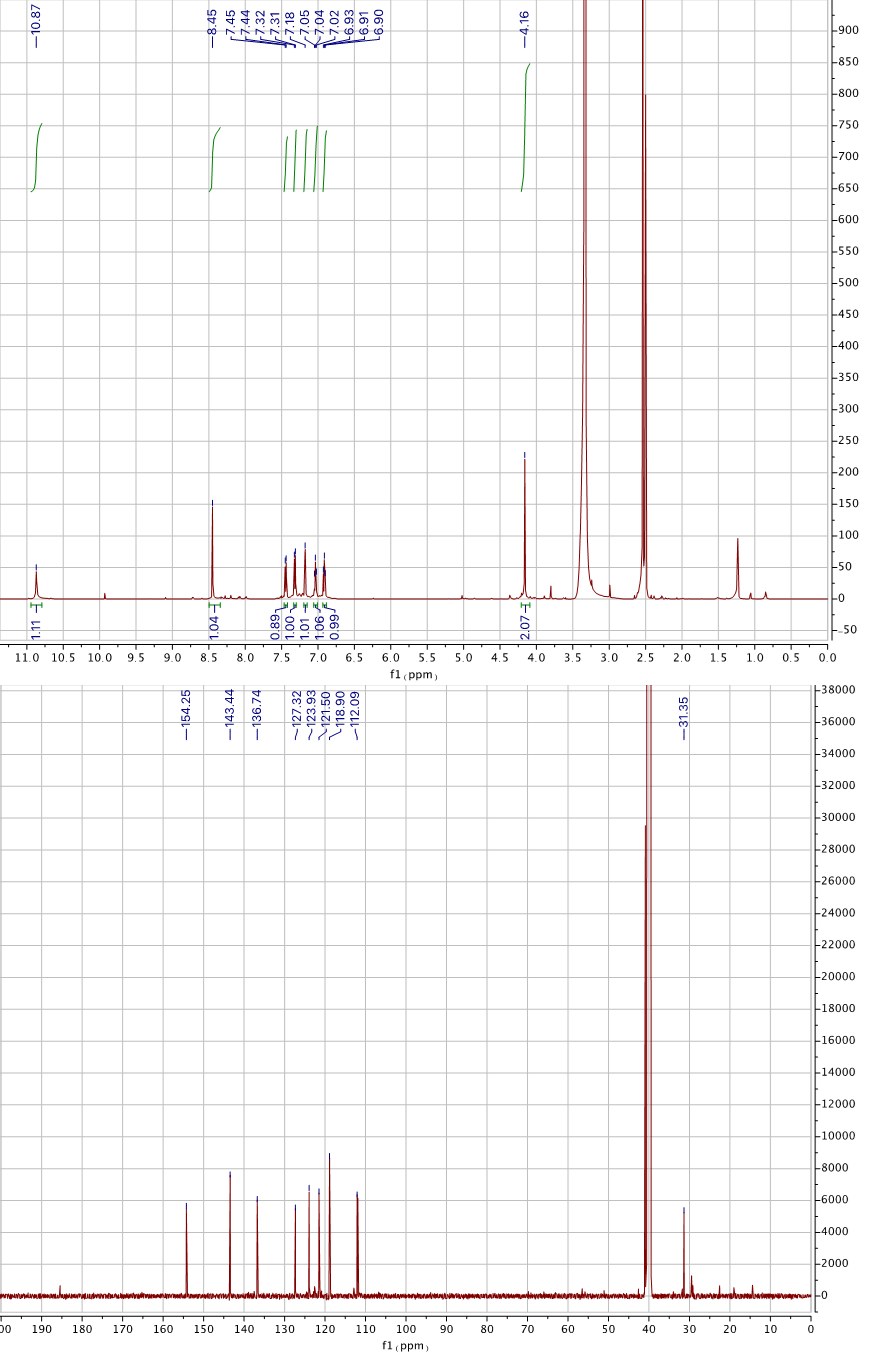

Supplement: FIG S3 [file msphere.00711-21-sf003.tif]

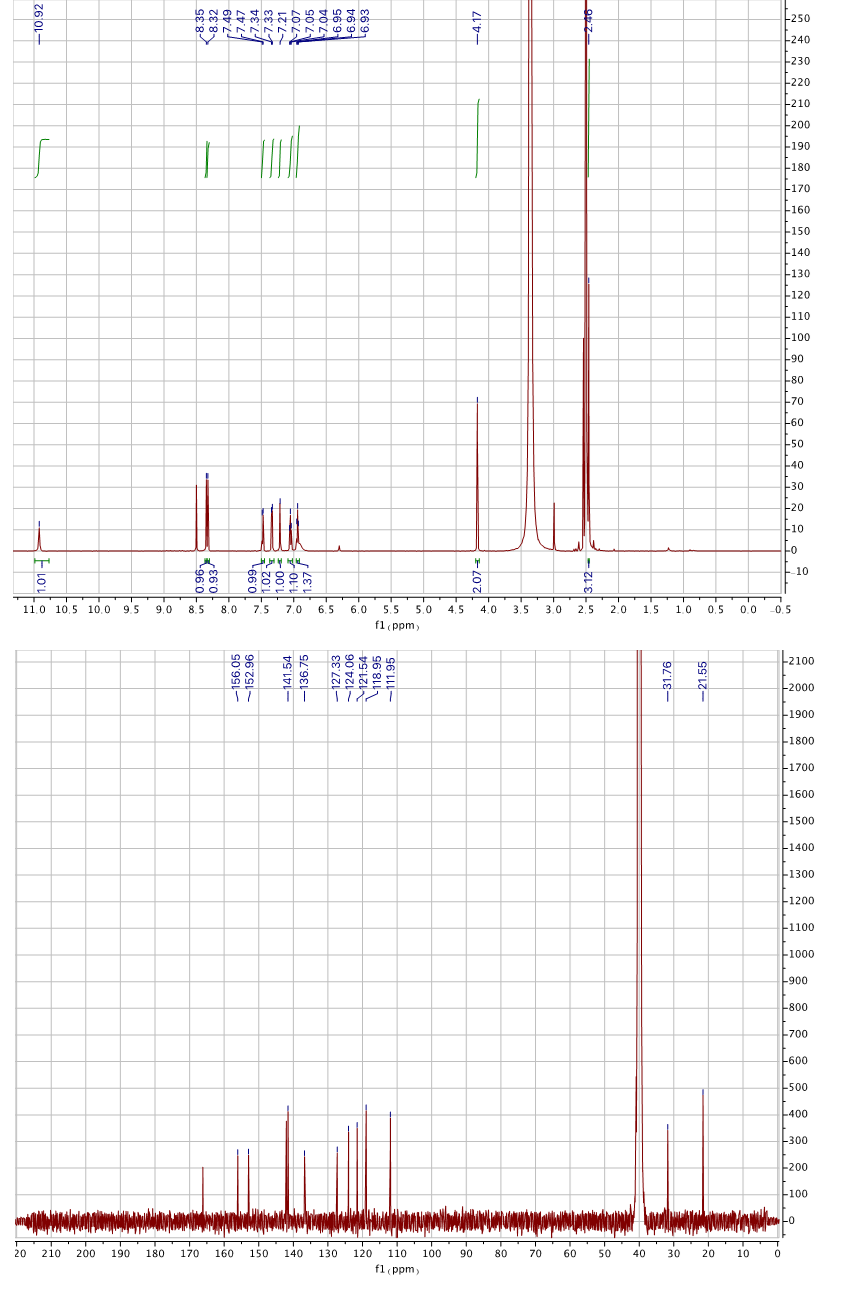

Supplement: FIG S4 [file msphere.00711-21-sf004.tif]

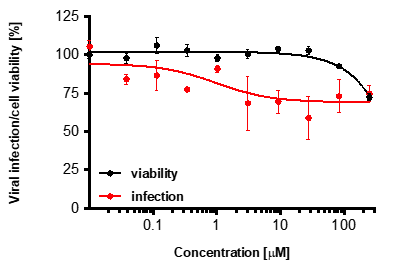

Supplement: FIG S5 [file msphere.00711-21-sf005.tif]

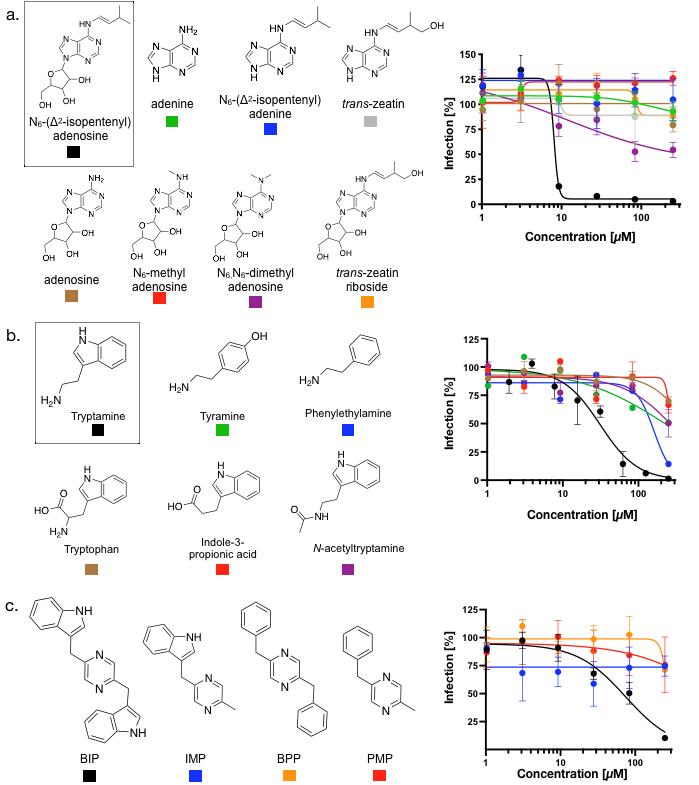

Supplement: FIG S9 [file msphere.00711-21-sf009.tif]

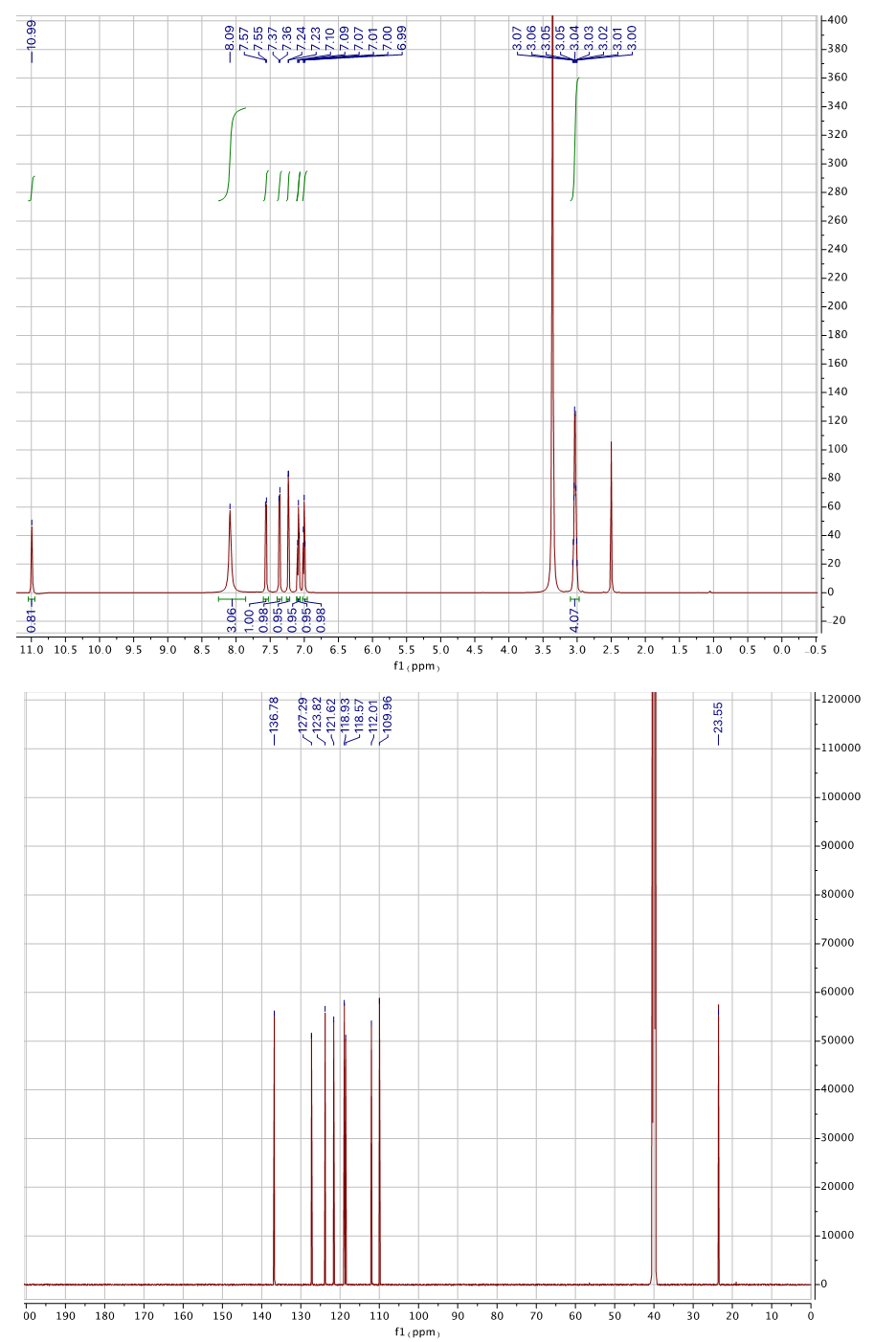

Supplement: FIG S6 [file msphere.00711-21-sf006.tif]

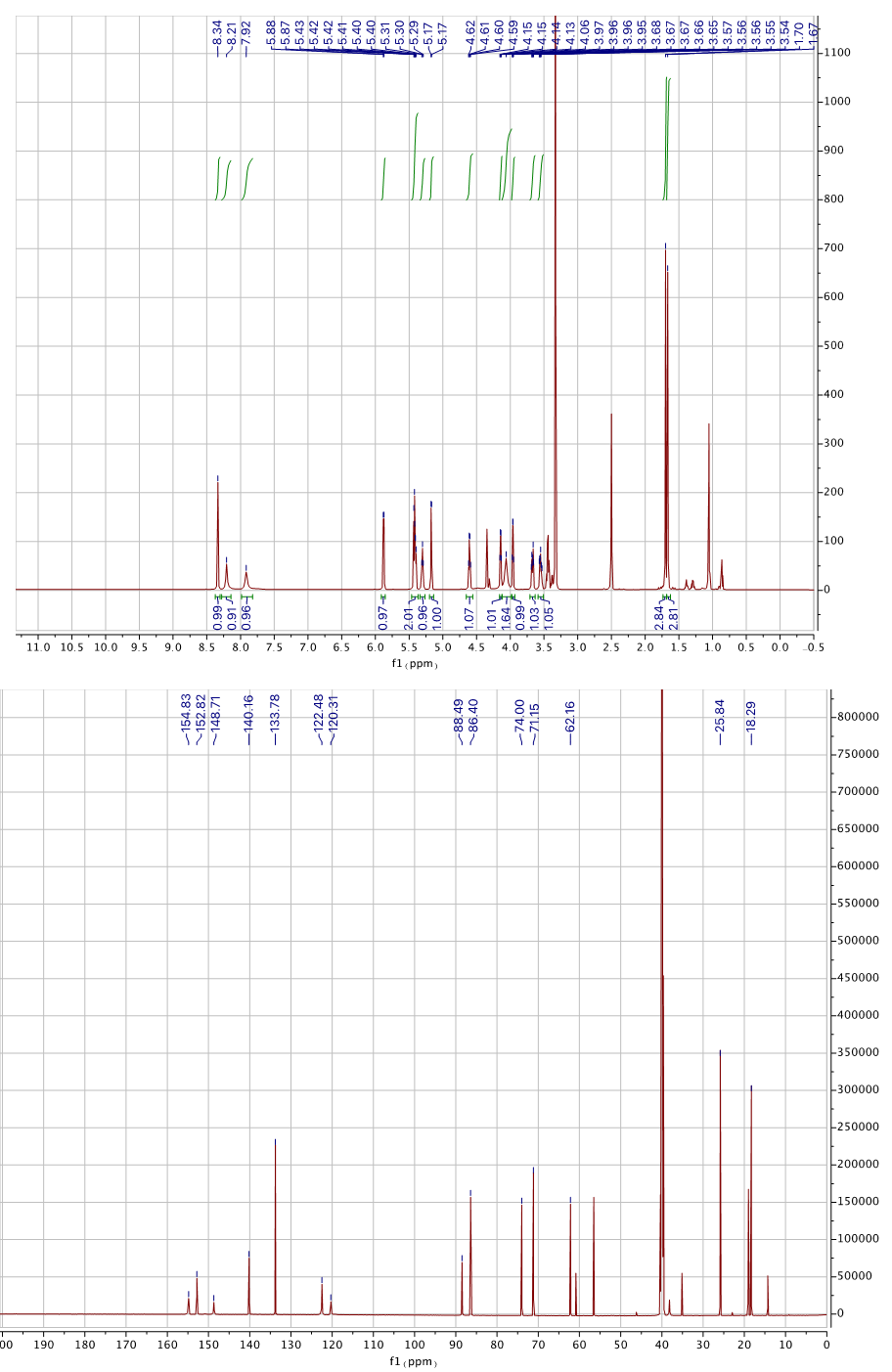

Supplement: FIG S7 [file msphere.00711-21-sf007.tif]

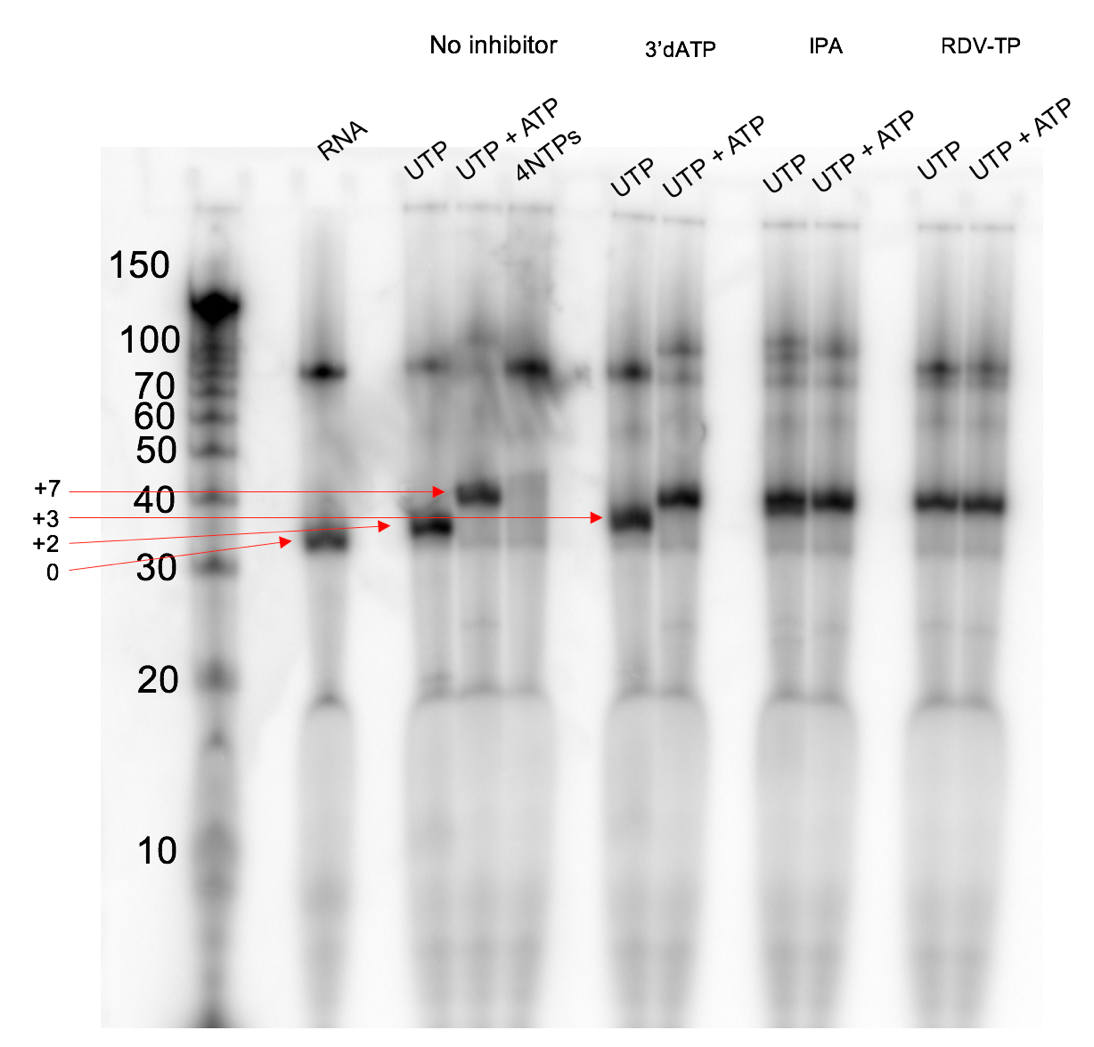

Supplement: FIG S8 [file msphere.00711-21-sf008.tif]
